# Timing of Food Intake Drives the Circadian Rhythm of Blood Pressure

**DOI:** 10.1093/function/zqaa034

**Published:** 2020-11-24

**Authors:** Dingguo Zhang, Jackson C Colson, Chunhua Jin, Bryan K Becker, Megan K Rhoads, Paramita Pati, Thomas H Neder, McKenzi A King, Jennifer A Valcin, Binli Tao, Malgorzata Kasztan, Jodi R Paul, Shannon M Bailey, Jennifer S Pollock, Karen L Gamble, David M Pollock

**Affiliations:** 1 Division of Nephrology, Department of Medicine; 2 Division of Molecular and Cellular Pathology, Department of Pathology; 3 Department of Psychiatry and Behavioral Neurobiology, University of Alabama at Birmingham, Birmingham, AL 35294, USA

**Keywords:** blood pressure, circadian rhythms, time-restricted feeding, sodium excretion, Bmal1

## Abstract

Timing of food intake has become a critical factor in determining overall cardiometabolic health. We hypothesized that timing of food intake entrains circadian rhythms of blood pressure (BP) and renal excretion in mice. Male C57BL/6J mice were fed ad libitum or reverse feeding (RF) where food was available at all times of day or only available during the 12-h lights-on period, respectively. Mice eating ad libitum had a significantly higher mean arterial pressure (MAP) during lights-off compared to lights-on (113 ± 2 mmHg vs 100 ± 2 mmHg, respectively; *P* < 0.0001); however, RF for 6 days inverted the diurnal rhythm of MAP (99 ± 3 vs 110 ± 3 mmHg, respectively; *P* < 0.0001). In contrast to MAP, diurnal rhythms of urine volume and sodium excretion remained intact after RF. Male *Bmal1* knockout mice (Bmal1KO) underwent the same feeding protocol. As previously reported, Bmal1KO mice did not exhibit a diurnal MAP rhythm during ad libitum feeding (95 ± 1 mmHg vs 92 ± 3 mmHg, lights-off vs lights-on; *P* > 0.05); however, RF induced a diurnal rhythm of MAP (79 ± 3 mmHg vs 95 ± 2 mmHg, lights-off vs lights-on phase; *P* < 0.01). Transgenic PERIOD2::LUCIFERASE knock-in mice were used to assess the rhythm of the clock protein PERIOD2 in ex vivo tissue cultures. The timing of the PER2::LUC rhythm in the renal cortex and suprachiasmatic nucleus was not affected by RF; however, RF induced significant phase shifts in the liver, renal inner medulla, and adrenal gland. In conclusion, the timing of food intake controls BP rhythms in mice independent of Bmal1, urine volume, or sodium excretion.

## Graphical Abstract

**Graphical Abstract:**
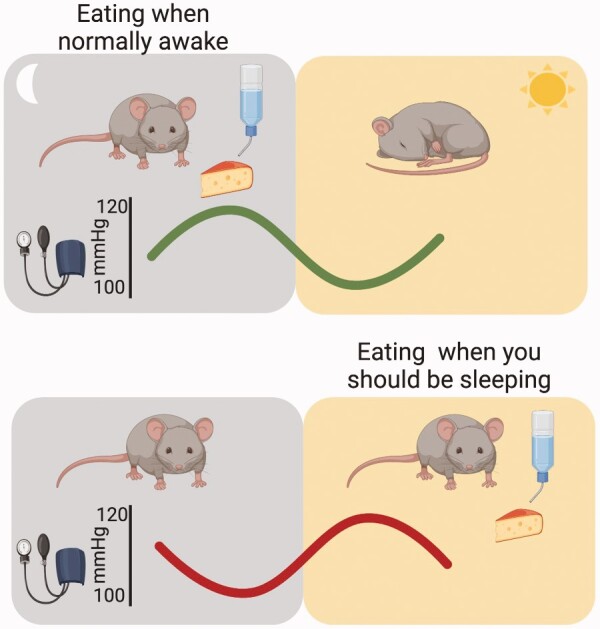

## Introduction

In healthy individuals, nighttime blood pressure (BP) is 10%–20% lower than the average daytime BP, a phenomenon described as BP dipping.^1^ Loss of BP dipping is associated with higher cardiovascular risk as well as more severe end-organ complications among hypertensive individuals. Therefore, nighttime BP is considered an independent indicator for BP management.^2^ Although mechanisms underlying BP dipping remain to be fully elucidated, many studies indicate that the capacity of the kidney to excrete sodium is a key factor.^3^^,^^4^ The kidney is critical for long-term BP regulation through control of sodium excretion and fluid–volume homeostasis,^4^^,^^5^ but whether renal handling of sodium accounts for the 24-h BP rhythm is not known.

It has long been appreciated in humans and animal models that kidney function follows a diurnal rhythm independent of meal timing and sleep.^6^^,^^7^ However, we have only recently begun establishing circadian mechanisms of fluid–electrolyte homeostasis. Microarray data have shown that roughly 20% of the genes expressed in the kidney follow a circadian pattern. These include a large number of genes related to electrolyte transport that are directly regulated by the circadian molecular clock.^8^ At its most simplified form, the circadian clock system consists of core circadian genes that form a transcription–translation feedback loop.^9^ The circadian proteins BMAL1 and CLOCK heterodimerize and bind to E-box elements of the circadian genes *Period (Per)* and *Cryptochrome (Cry)* to initiate transcription. The negative arm of the loop consists of PER and CRY proteins translocating into the nucleus and inhibiting the transcriptional activity of CLOCK-BMAL1 promoters on the E-box. Several laboratories have described the BP phenotypes in rodents lacking these main circadian genes.^10^^–^^11^ Of note, global genetic deletion of *Bmal1* in mice resulted in the loss of a diurnal BP rhythm along with many other circadian phenotypes.^13^ Many tissue-specific *Bmal1* knockout mouse models have also been generated to determine the mechanism for the arrhythmic BP phenotype observed in global *Bmal1* knockout mice^14^^–^^16^; however, the complete abolishment of BP rhythm was not seen in any of the tissue/cell-specific models. For example, Gong et al. showed that loss of *Bmal1* in smooth muscle cells significantly reduced, but did not eliminate, the day–night difference of BP.^17^

Light exposure is one of the most important environmental cues (zeitgeber or time giver) and maintains coordination via the suprachiasmatic nucleus (SCN) in the hypothalamus. Many circadian rhythms are maintained without light although with an intrinsic period length that is close to, but not exactly, 24 h.^18^ Light is one of the most important cues (zeitgeber or time giver) for synchronizing the internal circadian system with the environment by phase shifting the central pacemaker in the SCN in the hypothalamus. Circadian rhythms in peripheral organs are entrained by environmental factors other than light^19^ such as timing of food intake. Recent evidence suggests that time-restricted feeding (RF), such as intermittent fasting, provides beneficial cardiometabolic effects in humans. Sutton et al. carried out an early-time RF protocol (6 + 6 h feeding window including dinner before 03:00 pm) in prediabetic individuals.^20^ They found that 5 weeks of early-time RF lowered both daytime systolic and diastolic BP without reducing body weight. Moreover, Wilkinson et al. showed that a 10-h time RF protocol could reduce BP in obese humans.^21^ The circadian rhythm of BP was not addressed in these or other studies of time RF, and it is therefore important to understand how timing of food intake plays a role in diurnal BP regulation. In this study, we hypothesized that timing of food intake is a key factor in regulating circadian BP rhythms. Our approach was to misalign food availability with the normal light–dark cycle in otherwise healthy mice. We further investigated whether any effects of food intake on BP are dependent on the molecular clock by using both wild type (WT) and global *Bmal1* knockout (*Bmal1* KO) mice as well as PERIOD2::LUCIFERASE mice.

## Methods

### Animals

All animal studies were conducted with the approval of the University of Alabama at Birmingham Institutional Animal Care and Use Committee in accordance with the National Institutes of Health Guide for the Care and Use of Laboratory Animals. Male global *Bmal1* knockout mice (Bmal1KO) and WT littermate control aged 10–14 weeks old were purchased from the Jackson Laboratory (cat# 009100; Bar Harbor, ME). The helix-loop-helix domain within exon 4 and all of exon 5 were replaced with a neomycin resistance gene cassette. Male C57BL/6J mice aged 10–12 weeks old were purchased from The Jackson Laboratory (stock number 000664) and used for glomerular filtration rate (GFR) measurements, constant darkness BP experiments, and 24-h plasma collections. Animals were kept in a room with either 12 h light/12 h dark conditions or in 24 h constant darkness when indicated. Male *Per2^Luc+/−^* knock-in mice (16–60 days old) backcrossed to C57BL/6 for 12 generations were used to measure *Per2*-driven luminescence (PER2::LUC).

### BP and Heart Rate Measurements by Telemetry

Telemetry transmitters (TA11-PAC10, Data Science International, St. Paul, MN) were implanted into the right carotid artery of mice as previously described to measure BP, heart rate (HR), and activity.^16^ Mice were allowed to recover from surgery at least 10 days prior to starting any experiment. Telemetry data were recorded at 2000 samples/s in 3-min bins every 20 min.

### Feeding Intervention and Metabolic Cage Study

Mice were maintained in metabolic cages designed for urine collection (Fisher Scientific, Tecniplast™). Gel diet (Micro stabilized Rodent Liquid Diet, TestDiet, St. Louis, MO) containing 6.3% agar was used throughout the study to prevent contamination of urine by regular pellet chow. Mice were given 3 days to acclimate to metabolic cage and gel diet prior to feeding intervention. During RF, mice were given 3 days of ad libitum feeding followed by 6 days of manual reverse feeding (RF), where food was available only during the 12 h lights-on period. Twelve-hour food and water intake as well as urine output was monitored corresponding to the lights-on and lights-off time periods.

### Constant Darkness Experiment

To conduct experiments without a light zeitgeber, mice were kept in a designated and isolated room within the animal facility where the lights were off for the duration of the study. Mice were acclimated to total darkness for a period of at least 3 weeks prior to the experiment. After baseline telemetry experiments of at least 3 days, mice were divided into three groups. Mice in the ad libitum feeding group always had food available, whereas separate animals had limited food availability corresponding to either 07:00 am–07:00 pm clock time (similar to RF group) or 01:00 pm–01:00 am clock time for a 6-day period. The latter group was designated as the offset fed (OF) group. The circadian time (CT) of these groups was determined by the activity onset (CT 12) and found to occur at 06:47 pm (on average, ±SD of 28 min) for the RF group and at 05:55 pm (±24 min) for the OF group. The phases of the telemetry measures were calculated using the cosinor fit function in ClockLab (Actimetrics, Wilmette, IL) on the last 4 days of RF. Circular statistics were analyzed and Rayleigh plots made using Orianna 4 (Kovach Computing Systems, Anglesey, Wales).

### Urinary Measurements

An atomic absorption spectrometer (Analyst 200, Perkin Elmer, Waltham, MA) was used to determine urinary sodium and potassium concentrations. Urinary endothelin-1 (ET-1) concentrations were determined by ELISA (QuantiGlo ET-1 Kit, R&D Systems, Minneapolis, MN). Reagents for measuring urinary aldosterone concentration were generously provided by Drs. Elise and Celso Gomez-Sanchez at University of Mississippi Medical Center.^22^ Briefly, a 96-well plate was coated with mouse gamma globulin overnight and washed 3 times prior to adding goat-anti-mouse IgG. The plate was washed three times after a 30-min incubation. Afterward, 50 µL Aldo A2E11 Integra antibody (1:75 000 dilution) was added to each well along with 50 µL standards or samples and 50 µL aldo-3CMO-biotin. After a 2 h incubation, the plate was washed and avidin horseradish peroxidase was added. Chromogenic substrate tetramethylbenzidine was added and plate was put in a dark room for a 30-min incubation prior to plate reading.

### Plasma Hormone and Electrolyte Measurement

Plasma samples were collected into heparin-coated tubes at six time points (ZT 1, ZT 5, ZT 9, ZT 13, ZT 17, ZT 21) throughout a 24-h period. Plasma leptin concentrations were determined by ELISA (mouse leptin ELISA kit, 90030, Crystal Chem, IL). Plasma insulin concentrations were determined by ELISA (Ultra Sensitive Mouse Insulin ELISA Kit, 90080, Crystal Chem, IL). Plasma IGF-1 concentrations were determined by ELISA (Mouse/Rat IGF-I/IGF-1 Quantikine ELISA Kit, MG100, R&D Systems, MN). Plasma corticosterone concentrations were determined by ELISA (corticosterone ELISA Kit, K014-H1, Arbor Assays, MI). Plasma sodium, potassium, and chloride levels were measured using the Abbott point of care i-STAT Alinity system.

### Measurement of GFR

GFR was measured in a separate cohort of age-matched C57BL6/J mice under ad libitum or RF. Half of the mice in each of the two dietary regimens were placed in a regular 12:12 light:dark room with lights on (ZT 0) at 07:00 am. The other half of each group was entrained to a reverse lightroom with lights on (ZT 0) at 09:00 pm at least 3 weeks prior to study. Transcutaneous measurement of fluorescein isothiocyanate (FITC)–labeled sinistrin was used to measure GFR at ZT 4 and ZT 16^23^^,^^24^ (Transdermal GFR monitor; MediBeacon GmbH; FITC-sinistrin: 0.15 mg per gram of body weight; Fresenius Kabi Austria GmbH).

### Organotypic Cultures and PER2::LUC Bioluminescence Assays

*Per2^Luc+/−^* knock-in mice^25^ were euthanized after ad libitum or RF protocol by rapid decapitation. Coronal slices (250 µm) of the hypothalamus were cut on a vibroslicer and the SCN was carefully dissected under a dissection microscope in cold sampling media (HBSS, 14175-103 Gibco™ [Sigma-Aldrich, Carlsbad, CA], supplemented with 25 U/mL streptomycin/penicillin, NaHCO_3_ [7.5%, Sigma-Aldrich, St. Louis, MO], and 1.0 M HEPES). Kidney cortex and inner medulla were dissected by hand with a scalpel blade. Liver sections from each animal were dissected from the outer area of the large lobe. Whole adrenal glands, liver, and kidney tissues were rinsed with cold sampling media, and transferred to a mesh macroporous filter (Spectrum Laboratories, Inc., Rancho Dominguez, CA). Each tissue section was placed separately into a 35 mm cell culture dish containing 1.5 mL of recording media supplemented with putrescine (0.1 mM; MP Biomedicals, LLC, Solon, OH) and insulin (10 ng/mL; Roche).^26^ Tissues were then transferred to culture membranes (Millipore, Billerica, MA) in 35-mm culture dishes with 1.0 mL of DMEM (90-013-PB; Cellgro, Manassas, VA) supplemented with 4.5 g/L glucose, 1.0 M HEPES, 25 U/mL penicillin/streptomycin, 2% B27, and 0.1 mM beetle luciferin (Promega™ Corporation, Madison, WI). Tissue cultures were maintained at 36°C in an incubator. Bioluminescent rhythms of PER2::LUC were acquired with a LumiCycle^®^ apparatus and analyzed with LumiCycle^®^ data analysis software (Actimetrics Wilmette, IL).

### Statistical Analysis

All results are expressed as means ± SE. Specific statistical tests are indicated in figure and table legends. In general, Student’s *t*-test was used to compare differences in the 24-h average of BP and HR, as well as the acrophase of PER2::LUC. Two-way ANOVA with Sidak’s multiple comparison was used to compare food intake, water intake, urine production, urinary sodium, and potassium excretion, ET-1, aldosterone, GFR, and plasma hormones. A probability of *P* < 0.05 was considered significant.

## RESULTS

### RF Inverts MAP But Not Renal Excretion in WT Mice

**Figure 1.**
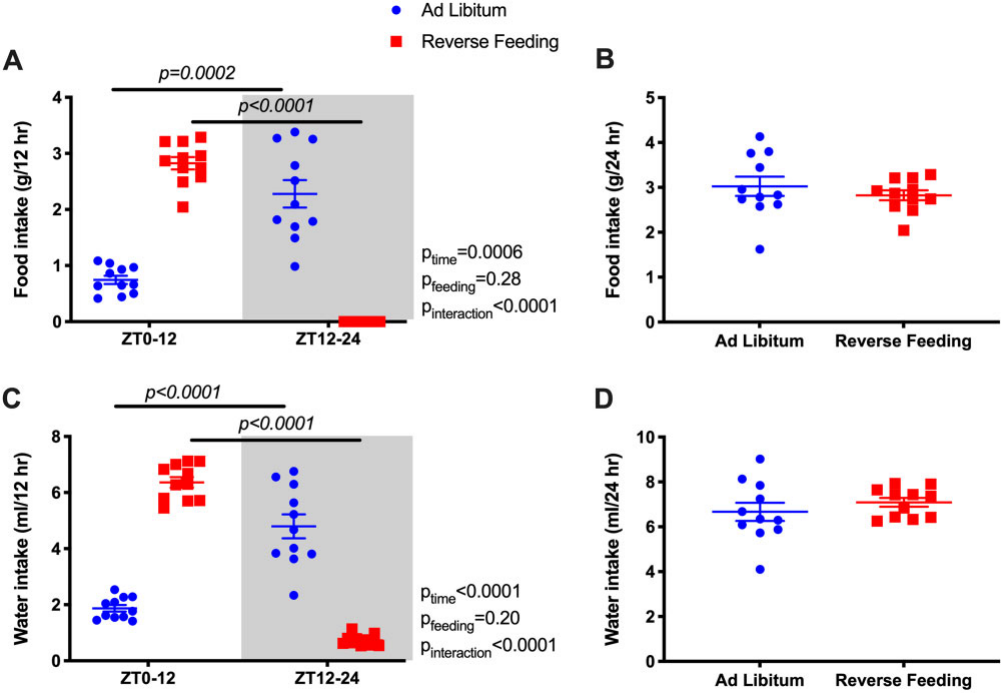
Food and water intake (average of the last 2 days) during ad libitum feeding and RF in WT mice. Data are presented as 12-h average (**A** and **C**) and 24-h average (**B** and **D**). Repeated measures two-way ANOVA was used in (A), (C), and indicated significant Feeding X Time interactions (*P* < 0.05; bars indicate significant post hoc comparisons). Student’s *t*-tests for unpaired data were used in (B), (D).

Food and water intake were monitored in 12-h increments from ZT0 to ZT12 and from ZT12 to ZT24 (Figure 1). During ad libitum feeding, WT mice had significantly higher food and water intake during active phase (ZT12–ZT24; Figure 1A and C). By design, food intake in the RF group was restricted to the inactive phase (ZT0–ZT12). However, total 24-h food intake was comparable between ad libitum and RF in WT mice (Figure 1B and D).

**Figure 2.**
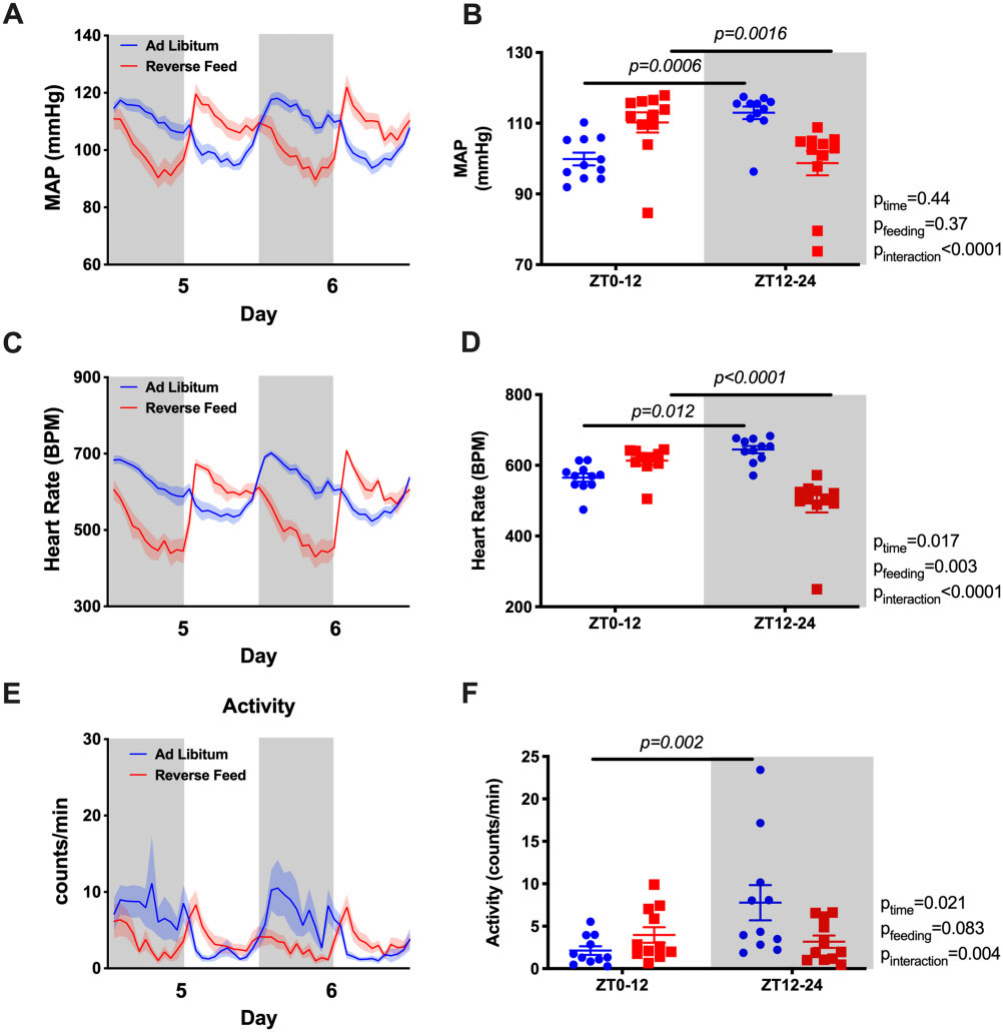
MAP and HR of the last 2 days during ad libitum feeding and RF in WT mice. Data are presented as 48-h trace with shaded areas indicating lights off (**A** and **C**) as well as 12-h average of the last 2 days (**B** and **D**). Repeated measures two-way ANOVA was used in comparing the 12-h average data and indicated significant Feeding × Time interactions (*P* < 0.05; bars indicate significant post hoc comparisons).

Under ad libitum conditions, mean arterial pressure (MAP) displayed a typical diurnal pattern when examined in hourly averages (Figure 2A) or in 12-h day–night averages (Figure 2B). However, RF resulted in an “inversion” of the MAP rhythm such that MAP was significantly higher during the lights-on phase and lower during the lights-off phase. The total 24-h average MAP remained unchanged (Figure S1A). Similarly, RF led to an “inversion” of the HR rhythm that was higher during the light-on phase and lower during the light-off phase (Figure 2C and D) with a decrease in 24-h average HR (Figure S1B). RF reduced the difference between lights-on activity counts and lights off activity counts over each 12-h period (Figure 2E and F). Similar to MAP, both SBP and DBP had a rhythm inversion caused by RF (Figure S6A and B).

**Figure 3.**
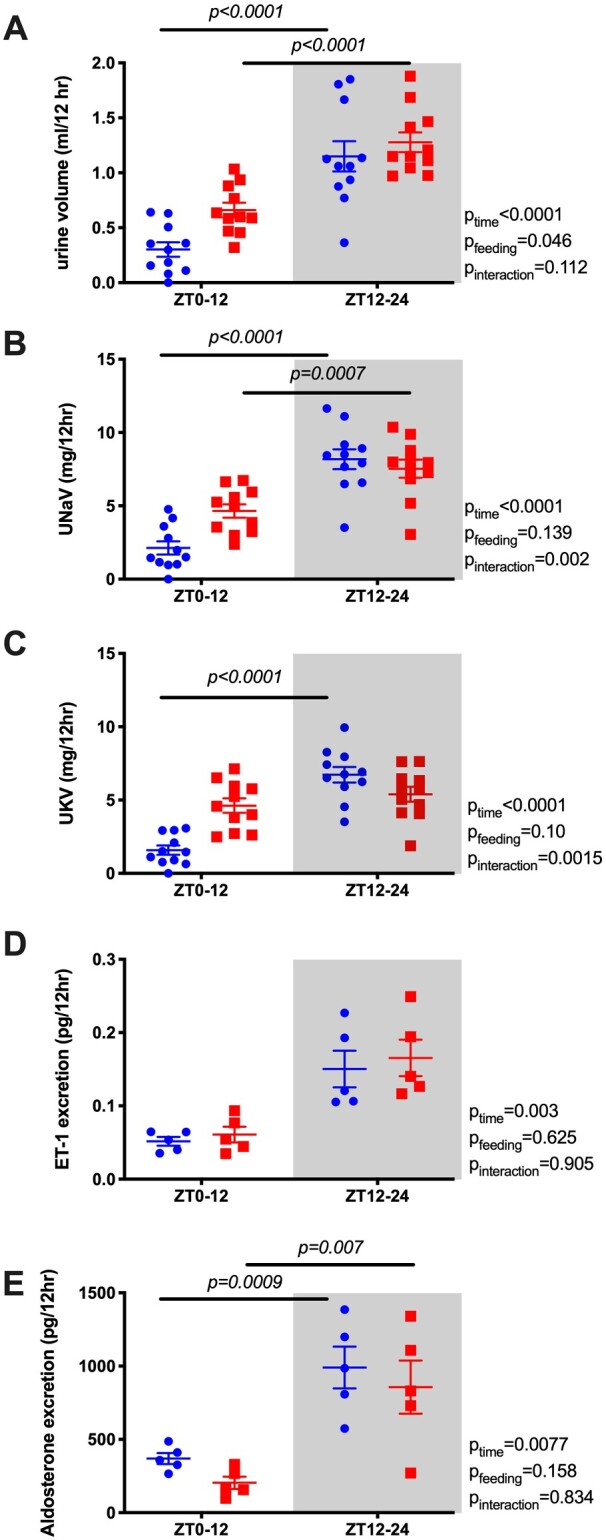
Urine volume (A), urinary sodium excretion (UNaV; B), urinary potassium excretion (UKV; C), urine excretion of ET-1 (D), and aldosterone (E) during ad libitum feeding and RF in WT mice. Data are presented as 12-h average of the last 2 days. Repeated measures two-way ANOVA indicated significant Feeding × Time interactions (*P* < 0.05; bars indicate significant post hoc comparisons).

Our original prediction was that the diurnal pattern of renal water and electrolyte excretion was driven by the BP rhythm. However, the diurnal pattern of urine volume (Figure 3A) and sodium excretion (Figure 3B) did not change or flip despite reversed food intake and MAP rhythms. In contrast, the normal day–night potassium excretion rhythm was absent in RF mice (Figure 3C). We also examined urinary excretion of two important hormonal regulators of sodium excretion, ET-1 and aldosterone. Both ET-1 and aldosterone normally follow a diurnal excretion pattern during ad libitum feeding, which was not significantly changed by RF (Figure 3D and E). RF led to an increase in 24-h urine volume, but there was no statistically significant difference in the excretion of sodium, potassium, ET-1, or aldosterone (Figure S2A–E).

### MAP and Renal Excretion Rhythms Are Independent of Bmal1

**Figure 4.**
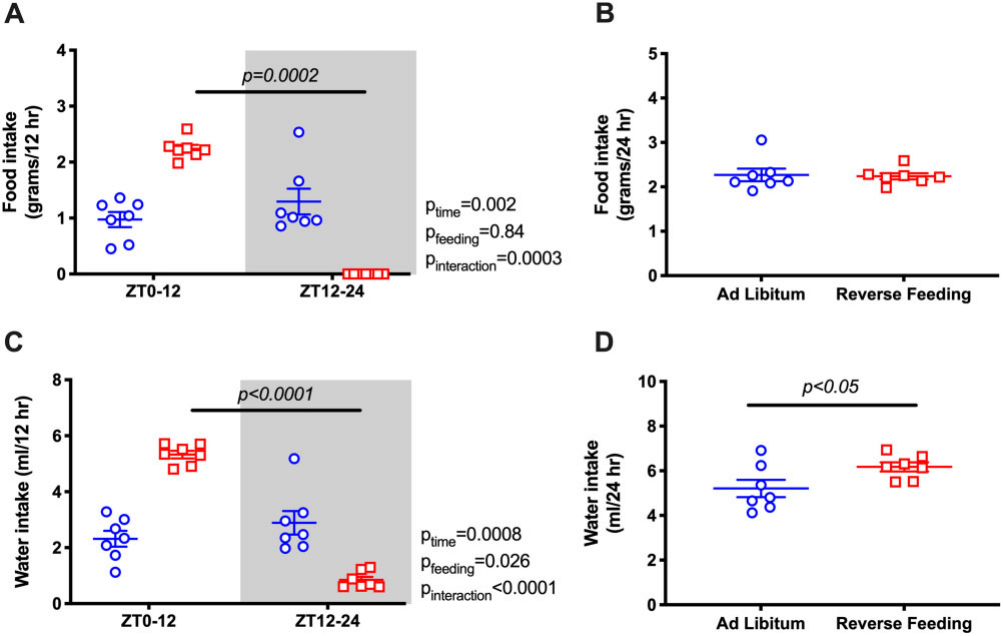
Food and water intake (average of the last 2 days) during ad libitum feeding and RF in Bmal1KO mice. Data are presented as 12-h average (**A** and **C**) and 24-h average (**B** and **D**). Repeated measures two-way ANOVA was used in (A) and (C), indicating significant Feeding × Time interactions (*P* < 0.05; bars indicate significant post hoc comparisons). Student’s *t*-test for unpaired data was used in (B), (D).

Under ad libitum conditions, Bmal1KO mice did not have a diurnal rhythm in food intake (Figure 4A) or water intake (Figure 4C). When food was restricted to ZT0-ZT12 in Bmal1KO mice, intake was increased during the 12-h feeding period, but 24-h food intake between ad libitum and RF remained similar (Figure 4B). Interestingly, RF led to a mild but statistically significant increase in 24-h water intake in Bmal1KO mice (Figure 4D) but not in WT mice (Figure 1D).

**Figure 5.**
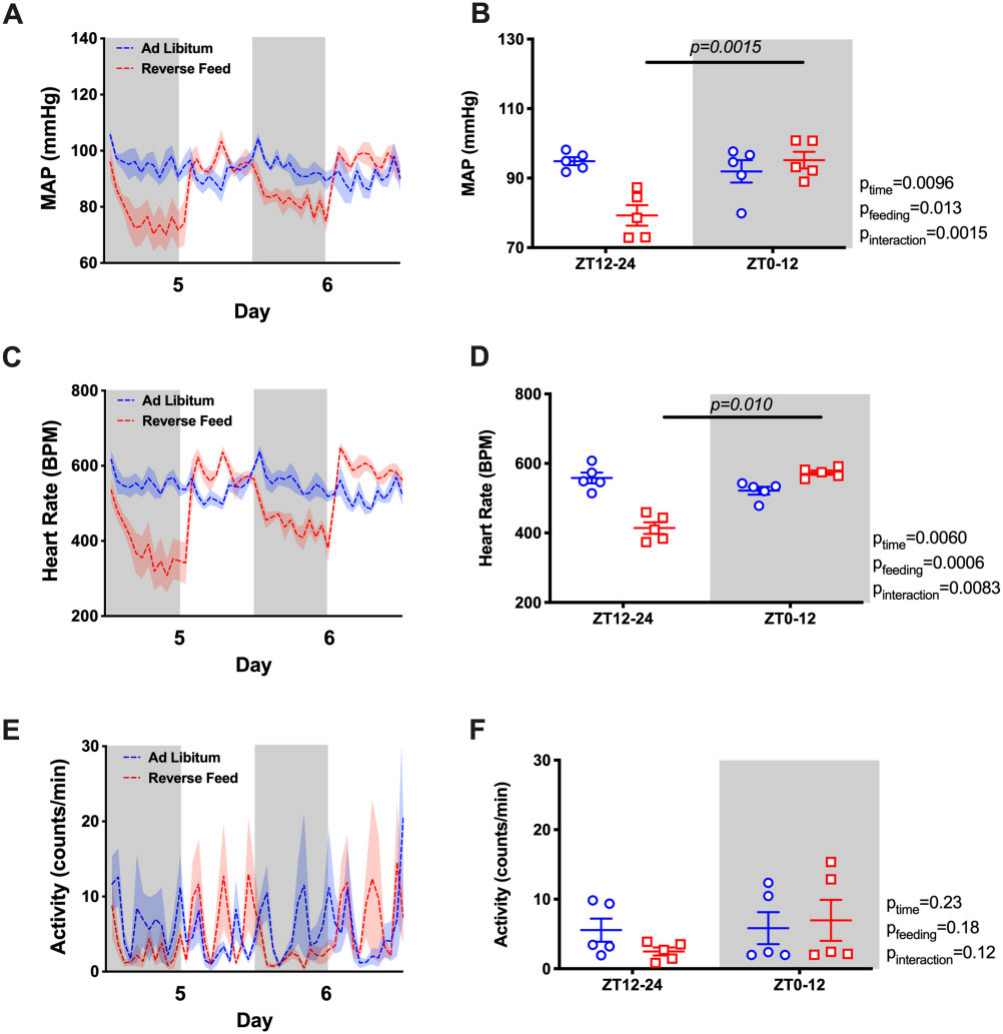
MAP and HR of the last 2 days during ad libitum feeding and RF in Bmal1KO mice. Data are presented as 48-h trace (**A** and **C**) as well as 12-h average of the last 2 days (**B** and **D**). Repeated measures two-way ANOVA comparing the 12-h average data indicated significant Feeding × Time interactions (*P* < 0.05; bars indicated significant post hoc comparisons).

As previously reported, Bmal1KO mice do not exhibit a circadian rhythm in MAP or HR when food and water are freely available the entire day.^13^ Remarkably, during the lights-on phase when food was available to the RF group, both MAP and HR were higher than during the light-off phase when food was withdrawn (Figure 5A–D). Similarly, SBP and DBP were higher during the lights-on phase (Figure S6C, 6D). RF did not change 24-h average MAP but led to a decrease in 24-h average HR (Figure S3A and B). For both groups, no differences in 12-h activity counts for lights-on versus lights-off phases were observed (Figure 5E and F).

**Figure 6.**
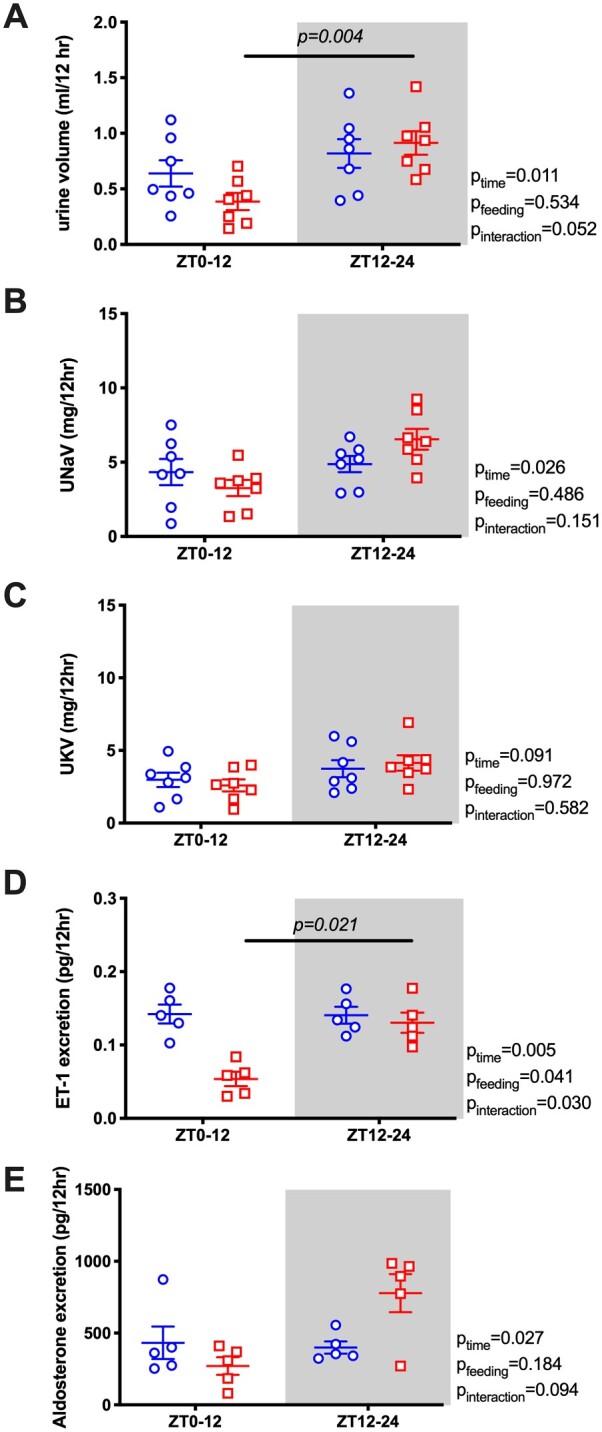
Urine volume (A), urinary sodium excretion (UNaV; B), urinary potassium excretion (UKV; C), urine excretion of ET-1 (D), and aldosterone (E) during ad libitum feeding and RF in Bmal1KO mice. Data are presented as 12-h average of the last 2 days. Repeated measures two-way ANOVA was used.

Bmal1KO mice under ad libitum conditions did not display a diurnal difference in urine output, sodium, potassium, ET-1, or aldosterone excretion, which parallels the loss of BP rhythm and diurnal food and water intake in these mice (Figure 6). After RF, however, diurnal rhythms in urine output, sodium, ET-1, and aldosterone excretion were established. It is important to note that this “re-established” diurnal rhythm of the Bmal1-deficient kidney was of the opposite pattern of MAP rhythm and followed the typical rhythm of the ad libitum WT mouse (higher during the active period). Interestingly, ad libitum fed Bmal1KO mice had overall higher 24-h ET-1 excretion than RF Bmal1KO mice suggesting that RF restored 24-h levels of ET-1 excretion but not 24-h urine volume or excretion of sodium, potassium, or aldosterone (Figure S4). Finally, urinary potassium excretion was not significantly changed by RF in the Bmal1KO mouse and remained essentially arrhythmic.

### RF Does Not Change GFR

**Figure 7.**
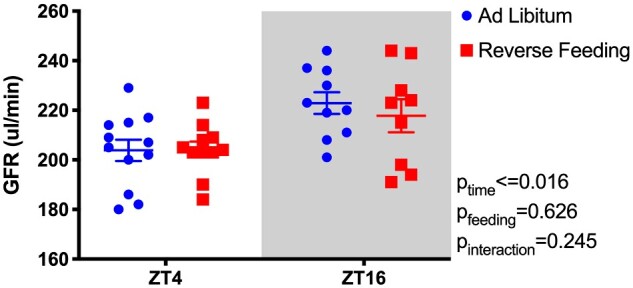
GFR of C57BL6/J mice at Day 6 of either ad libitum feeding or RF. GFR was measured at ZT4 and ZT16. Mixed effects two-way ANOVA reveals significant effect of Time, but no effect of Feeding or Time × Feeding interaction.

We further assessed the effect of RF on overall kidney function by measuring GFR in a separate cohort of C57BL/6J mice. As expected, GFR was significantly higher during the lights-off phase (ZT16, Figure 7), but there was no significant effect of RF nor was there a significant interaction between time of day and feeding regimen.

### Effect of RF on Plasma Hormones

**Figure 8.**
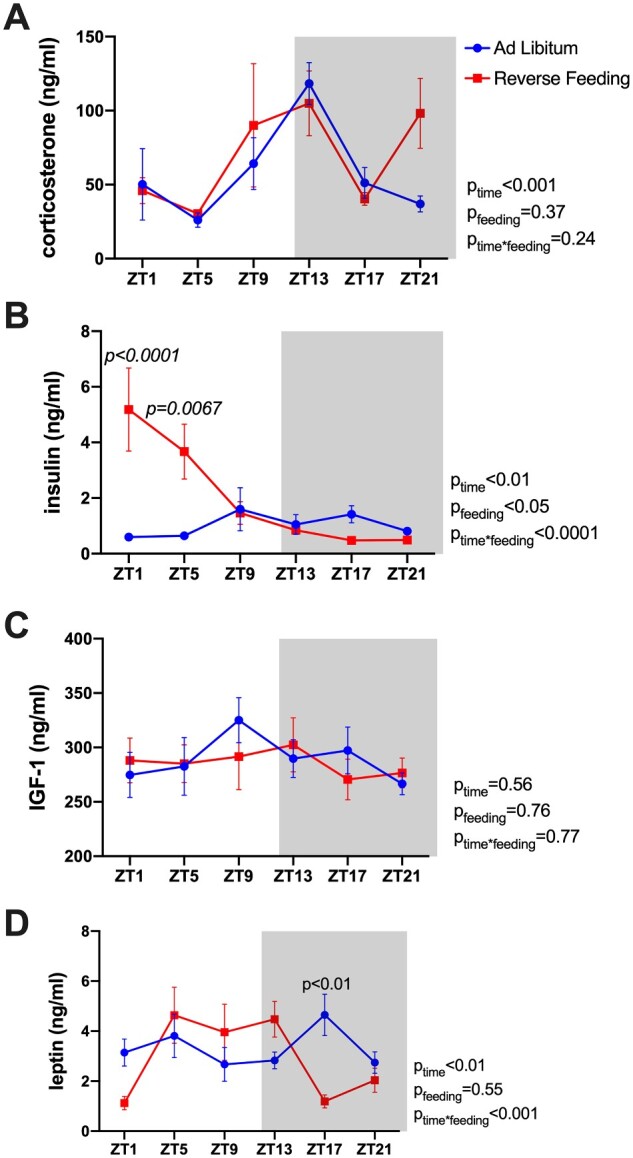
Plasma hormone levels of C57BL6/J mice at Day 6 of either ad libitum feeding or RF. Plasma levels of corticosterone (**A**), insulin (**B**), IGF-1 (**C**), and leptin (**D**) were measured from samples collected at six time points through a 24-h cycle. Repeated measures two-way ANOVA results are indicated on the right of each panel.

We next determined the effects of RF on the circulating levels of different circulating hormones in a separate cohort C57BL/6J mice given ad libitum or RF treatment maintained in a 12-h dark/light cycle. Specifically, individual mice were used to collect plasma over a 24-h period every 4 h for a total of six time points. Plasma was assayed for corticosterone, insulin, IGF-1, and leptin (Figure 8). Plasma corticosterone concentrations showed a significant time-of-day variation; however, RF did not have a significant impact. RF did not significantly change plasma IGF-1 levels. However, plasma insulin and leptin levels were significantly different between ad libitum and RF groups and tended to be higher during the inactive period in the RF mice compared to the ad libitum control mice. Plasma sodium and potassium levels were not affected by RF (Figure S5A and B). Similarly, there was no significant effect of time or feeding regimen on plasma chloride concentrations, although there was a significant interaction with plasma chloride being slightly elevated in the RF group during the day and slightly lower during the night (Figure S5C).

### RF Caused Differential Disruption of PER2::LUC Rhythm in Key Circadian Control Tissues

**Figure 9.**
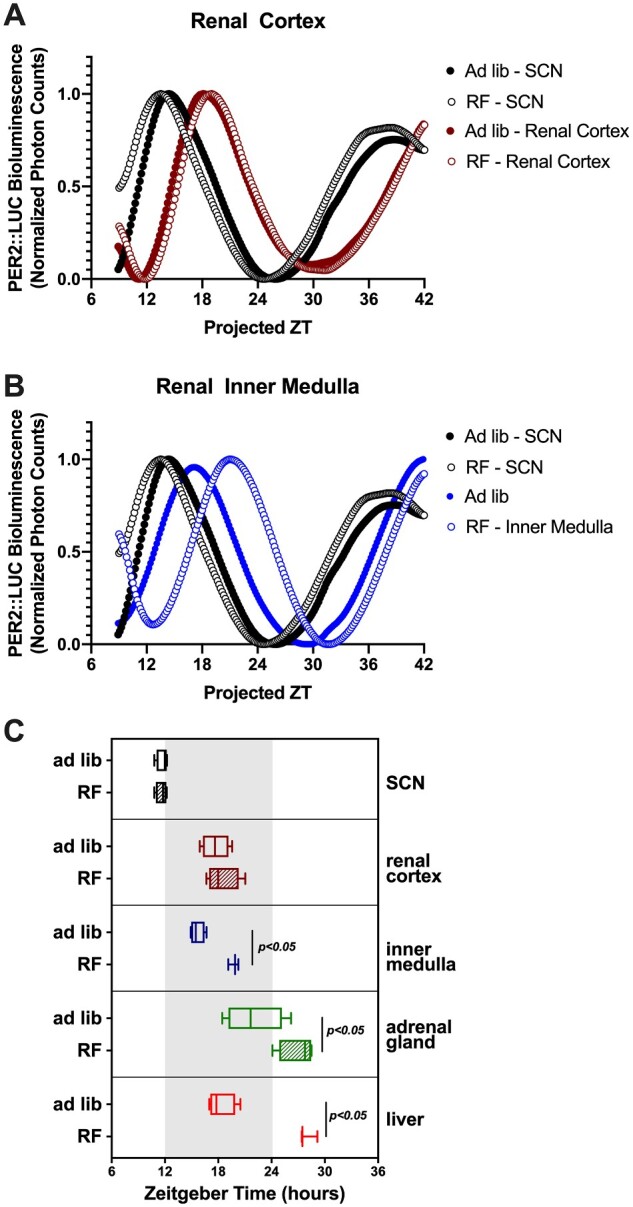
Lumicycle data of tissue cultures from *Per2^Luc+/−^* knock-in mice. Representative traces of bioluminescence in the SCN and renal cortex (**A**) and renal inner medulla (**B**) were shown. Acrophase (time of peak) of the SCN, renal cortex, inner medulla, adrenal gland, and liver were shown (**C**). Student’s *t*-test indicated significant phase differences between ad libitum and RF for inner medulla, adrenal gland, and liver (*n* = 3–4/group).

We used *Per2^Luc+/−^* knock-in mice to assess the phase of the rhythm of the clock protein PER2 (specifically, PER2::LUC) in the SCN (central circadian pacemaker), liver, renal cortex, renal inner medulla, and adrenal gland collected after ad libitum or RF diets (representative bioluminescence traces are in Figure 9A and B). The peak times of the PER2::LUC rhythm in the SCN and renal cortex were not significantly affected by RF (Figure 9C). However, in the liver, renal inner medulla, and adrenal gland, there were significant phase delays caused by RF (10 ± 1 h in the liver, 4 ± 1 h in the inner medulla, 5 ± 2 h in the adrenal gland; Figure 9C).

### Timing of Food Intake Controls Diurnal MAP Rhythm in Constant Darkness

**Figure 10.**
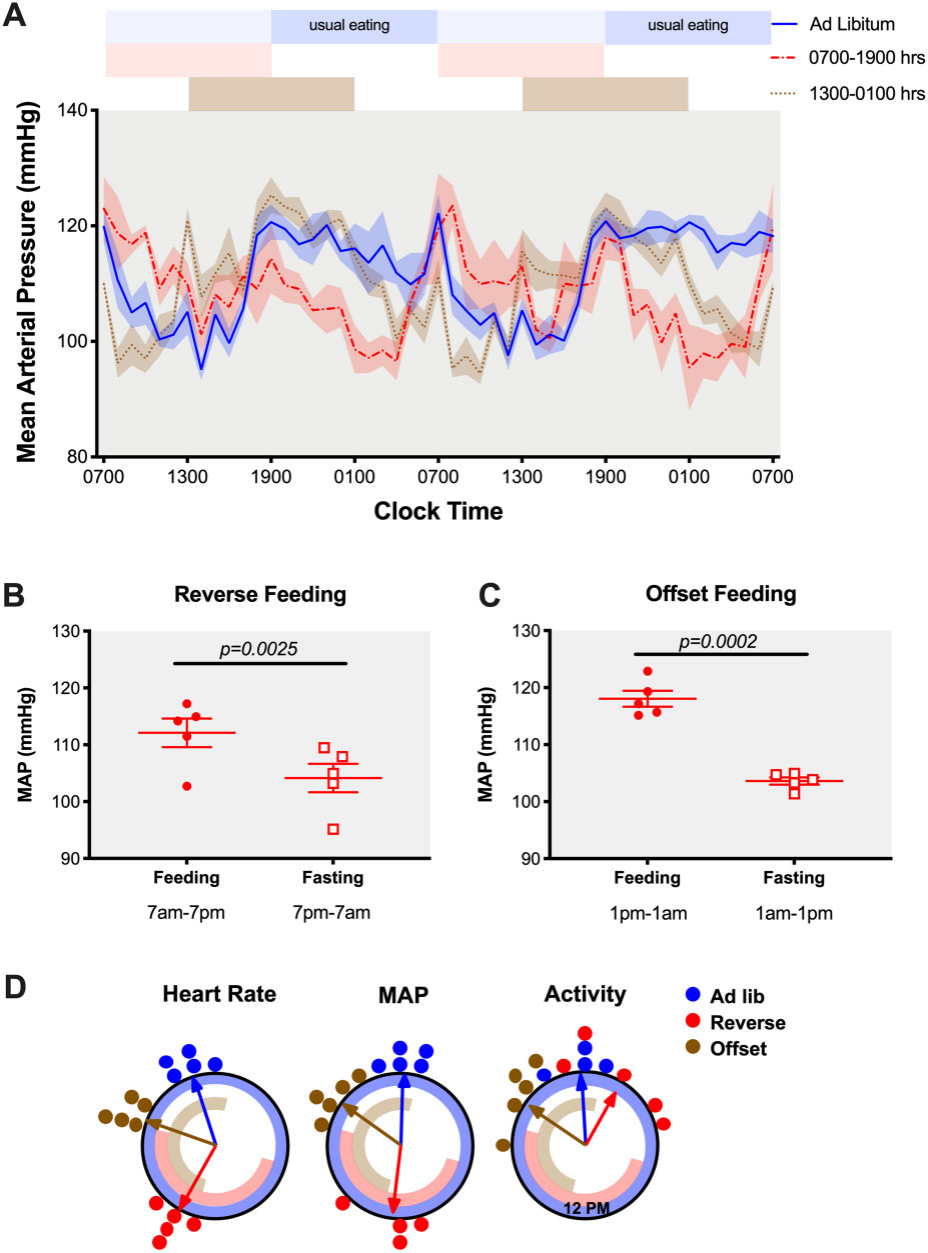
MAP of C57BL6/J mice at Day 5 and Day 6 of either ad libitum feeding, RF (07:00 am–07:00 pm feed), or offset feeding (01:00 pm–01:00 am feed) under constant dark conditions. Data were presented as 48-h trace (**A**) as well as 12-h averages of the feeding and fasting periods for RF group (**B**) and offset feeding group (**C**). Student’s *t*-test indicated significant fasting-feeding differences in the 12-h average MAP data for either feeding time. (**D**) Rayleigh plots for phase of peak HR, MAP, and activity from C57BL6/J mice during the last 4 days of ad libitum feeding, RF, or offset feeding. Circles represent the phase for individual mice and arrows represent the average phase for each feeding condition. Timing of food availability for each group is represented by the blue (ad libitum), red (RF), and brown (OF) bands inside the circle.

To determine whether RF impacted MAP rhythms by a direct effect of food timing alone or conversely by a food timing misalignment with the light–dark cycle, we acclimated a separate cohort of C57BL/6J mice implanted with telemetry transmitters in constant darkness and adjusted the time of food availability. In this experiment, we studied three separate groups, (1) ad libitum feeding, (2) RF feeding corresponding to clock time 07:00 am–07:00 pm (∼CT0 to CT12), and (3) a group in which we fed mice from clock time 01:00 pm to 01:00 am (OF, offset feeding, ∼CT8 to CT20). The rationale for adding this offset feeding group was to test if this intermediate misalignment (6 h instead of 12 h in RF group) in feeding time would cause corresponding changes in BP rhythm. The ad libitum group maintained a rhythmic MAP with the peak of MAP corresponding to the approximate normal time of food intake, ie, 00:20 am ± 32 min (where CT 12 was calculated to occur at 06:52 pm ± 06 min). RF and OF groups followed MAP rhythms with the highest BP readings corresponding to the individual feeding periods (12:26 pm ± 63 min and 08:29 pm ± 30 min, respectively, Figure 10A and D). Indeed, we calculated 12-h MAP averages from feeding and fasting periods in RF and OF groups and found that both RF and OF groups maintained significantly higher MAP when they had access to food, regardless of CT of food access (Figure 10B and C). The peak of HR was similarly aligned with the timing of typical food intake in the ad libitum group (10:46 pm ± 29 min) or timing of food availability for the RF and OF groups (01:53 pm ± 18 min and 07:36 pm ± 21 min, respectively, Figure 10D). When MAP and HR are plotted in alignment with when food is available in the RF and OF groups, we observed a similar increase around the time of food availability even though the sustained pattern during food availability is somewhat different between RF and OF (Figures S7D and E). Similar patterns can be seen for both systolic and diastolic BP as well as HR (Figure S7A–C). Overall locomotor activity was aligned with MAP and HR in the ad libitum and OF groups (peak times at 11:53 pm ± 41 min and 08:33 pm ± 53 min, respectively), but this did not hold true for the RF group (peak time at 02:12 am ± 92 min; Figure 10D and Figure S7D). Finally, close examination of locomotor activity allowed us to determine food anticipatory activity calculated as the percentage of total daily activity that occurred within a 3-h window prior to food availability. We can see that there was a significant increase in food anticipatory activity in the RF group (23.7 ± 2.0 counts) compared to the OF group (11.0 ± 1.7 counts, *P* = 0.0013) suggesting a time-of-day dependent effect (Figure S7F).

## Discussion

**Figure 11.**
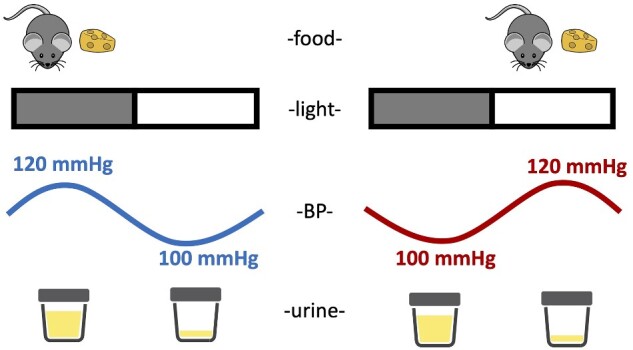
Schematic summary of the impact of meal timing on BP and urine production. When mice are fed ad libitum, both BP and urine volume follow diurnal rhythms that are high during the lights-off phase and low during the lights-on phase. When mice were reverse fed with food available only during the inactive period, the diurnal rhythm of BP is inverted; however, the diurnal rhythm of urine production remains intact.

The present study demonstrates the importance of timing food intake in mice on the circadian rhythms of BP (Figure 11). Loss of a circadian BP rhythm is frequently seen in certain patient populations, including patients with hypertension and chronic kidney disease.^27^^,^^28^ Clinical studies demonstrated correlations between arrhythmic BP and poor prognosis in different disease settings^29^^,^^30^; however, very little is known regarding causal mechanisms of this lack of rhythm. We observed that restricting food to the daytime in mice completely reversed the typical day–night BP rhythm. Thus, timing of food intake mediates the day–night rhythms in BP. The finding that daytime RF causes a reverse dipper BP phenotype has implications for a range of physiological and pathophysiological consequences that can occur in individuals such as shift workers, international travel as well as patients with chronic kidney disease and other disorders associated with circadian misalignments. Importantly, this rhythm is not controlled by the clock gene, *Bmal1*, and can override the light–dark cycle. Furthermore, we also demonstrate the profound uncoupling of renal solute handling and BP.

Previous studies in both humans and rodents have demonstrated beneficial effects of active period time RF such as BP reduction in the setting of metabolic syndrome.^20^^,^^31^ Unfortunately, the circadian aspect of BP was not discussed in these studies. Here, we presented a feeding model of circadian disruption and hypothesized that inactive-time RF would disrupt the BP rhythm and kidney function. We observed a complete inversion of the circadian BP rhythm, without any change in the 24-h average BP. This indicates that time of feeding drives the circadian rhythm of BP in mice and is in antiphase with the 12-h light/12-h dark cycle when food is restricted to the daytime. To determine if this light-cycle/feeding-cycle misalignment contributes to the diurnal BP pattern, we designed a separate experiment and fed mice at different CTs in a constant dark room. It is important to note that we acclimated mice to constant darkness conditions for 3 weeks before starting the RF protocol. Under these conditions, we observed that the highest BP readings were during the feeding period, regardless of the animal’s circadian phase. Taken together, these data indicate that food consumption is a more important driver or entraining signal of the BP rhythm than light.

Decades of research have confirmed that the kidney plays an essential role in regulating BP. Given the change in BP corresponding to food availability, we expected to see similar “inversions” in kidney excretory rhythms. However, in WT mice, diurnal rhythms of urine output and urinary excretion of sodium, ET-1, and aldosterone remained intact regardless of the time of food intake time. This observation led us to propose that the molecular clock in the kidney is maintaining diurnal excretory rhythms in synchrony with the central clock and independent of eating behavior. This raises the possibility that mistimed salt intake can prolong the time that sodium is being retained. Similar to what had been shown before,^32^ GFR in rodents is higher during the lights-off period compared to the lights-on period; however, RF did not have a significant effect on overall GFR or the diurnal pattern. This unchanged GFR can explain, at least in part, the intact renal excretory rhythm, despite inversed MAP. These observations led us to propose that the molecular clock in the kidney is maintaining diurnal excretory rhythms in synchrony with the central clock and independent of timing of food intake cues. Therefore, since sodium is a constituent of the food, we propose that mistimed salt intake can prolong the time that sodium is being retained.

We also observed that urinary potassium excretion rhythm was abolished during daytime food restriction with comparable levels between the light-on and light-off phase. Diurnal potassium rhythm is not well understood, but it has been suggested that the beneficial effects of potassium supplementation on BP were partly due to the resetting of potassium excretion rhythm, especially when the BP rhythm is lost due to salt-sensitive hypertension.^33^ This change in diurnal potassium excretion contrasts with the diurnal rhythm of aldosterone excretion that remained intact and in phase with the light–dark cycle. Microarray analysis on mouse kidneys found that the majority of known potassium-regulatory genes were expressed in a circadian-dependent manner, including genes that encode Na^+^/K^+^ ATPase, H^+^/K^+^ ATPase, Kir1.3, ROMK, and the BK channel.^34^ Of note, Salhi et al. showed that the circadian expression of H^+^/K^+^ ATPase is key to maintaining the circadian rhythm of both urinary K^+^ excretion and plasma K^+^ concentrations.^35^ Further experiments are needed to determine what accounted for the dissociation of potassium excretion with sodium and aldosterone. In general, these observations suggest that the potassium excretion is better able to align with intake and uncoupled from that with sodium excretion and water output suggesting the mechanisms responsible for acclimating to intake are more efficient for potassium.

We performed additional experiments in *Per2^Luc+/−^* knock-in mice to assess the impact of the RF protocol on the timing of clock gene rhythms in the central (SCN) clock compared to the kidney and other key tissues. As has been reported previously,^36^^,^^37^ RF had no effect on the phase of PER2 in the SCN. Consistent with the changes in renal function, PER2::LUC peak phase remained unchanged in the kidney cortex as well. However, there was a significantly later phase in the inner medulla and adrenal gland, indicating a differential effect of RF on these tissues. Our group has previously shown a differential effect of dietary salt on clock gene rhythms between the renal cortex and renal medulla with the latter being phase shifted by high salt diets.^38^ Importantly, we also observed a phase shift in the liver roughly on the order of 10-plus h indicating that the liver clock is highly responsive to eating behavior.^36^

Considerable evidence has accumulated over the past several decades suggesting that a range of metabolic hormones impact BP, especially in hypertension. Many of these factors display circadian rhythms, and so we measured circulating levels of several key hormones. We were surprised that RF did not significantly affect corticosterone levels, by which we interpret that the inverted BP rhythm we observed is not driven by corticosterone or the stress of RF. However, RF had a fairly clear impact on the time-of-day pattern of plasma insulin and leptin. The general pattern of these hormones followed the expected pattern during ad libitum feeding.^^39–42^^ At ZT 1, insulin levels of mice in the ad libitum group were quite low at 0.58 ng/mL. RF caused a 9-fold increase in plasma insulin concentration at this time point. Further research is needed to determine whether this insulin surge over time would contribute to the development of metabolic dysfunction. Food consumption stimulates increases in plasma leptin, and so it was not surprising that we observed RF-induced increased plasma leptin during the day compared to night. The degree to which this change in leptin contributes to diurnal BP patterns needs to be investigated.

Our observations in the Bmal1KO mice further confirmed our hypothesis that the molecular clock drives renal excretory function independent of the timing of food intake. BMAL1 is an essential transcription factor that is a component of the cellular molecular function. We conducted additional RF experiments using Bmal1KO mice because prior studies with this mouse model reported a lack of a BP rhythm.^13^ We reasoned that this lack of BP rhythmicity is due to eating behavior. As opposed to most mouse models, Bmal1KO mice do not have a diurnal food intake behavior with ad libitum feeding. Interestingly, in Bmal1KO mice, a circadian BP rhythm was established after RF, which is direct evidence for external factors overriding the BP effects of central clock loss. This was largely due to a drop in BP when the animals were fasting consistent with a lower overall BP noted in other clock gene knockout animals including the Bmal1 KO rat.^13^^,^^16^^,^^43^^,^^44^ These results suggest that follow-up studies will be needed to more specifically determine how the loss of Bmal1 causes the observed changes in eating behavior and the BP phenotype. Many of the other clock gene-specific KO mice have abnormal BP rhythms and so we will also need to determine the extent to which feeding behavior is driving those phenotypes, if any. Bmal1KO mice did not exhibit a diurnal pattern of water and electrolyte excretion under ad libitum conditions, which paralleled their lack of a BP rhythm. Although this may be interpreted that a loss of BMAL1 abolishes the functionality of the kidney clock, a kidney excretory rhythm was re-established in a “normal” pattern when food was restricted to the daytime. In other words, the kidney excretory rhythm was higher during the active period and low during inactive period, despite their RF pattern. This was an unexpected finding but suggests that eating or some physiological response to eating, provides a zeitgeber that allows rhythmicity in renal excretion, albeit with an offset phase. Loss of Bmal1 in the SCN results in an arrhythmic central clock, and therefore, most aspects of physiological activities become arrhythmic. However, when other environmental factors were forced onto the Bmal1KO mice, the intrinsic clock of certain peripheral organs, such as the kidney, entrain to these external cues, compensating for adverse phenotypes through pathways that do not involve Bmal1. Work from others has shown that physiological responses can be achieved in peripheral organs when Bmal1 is absent.^45^^,^^46^ Our laboratory recently showed that Bmal1 in the kidney collecting duct does not contribute to BP rhythm regulation, nor the kidney excretory rhythm.^16^ Crislip et al. recently reported that loss of Bmal1 in the entire distal nephron also did not alter the rhythm of BP when on an ad libitum diet.^43^ Work from Firsov and colleagues has also shown that *Bmal1* in different parts of the kidney (renal tubular cells, renin-secreting cells, and podocytes) also does not appear to directly regulate the circadian pattern of BP.^14^^,^^15^^,^^32^ These data suggest that Bmal1 in the kidney does not contribute to BP rhythm regulation. Aside from the global Bmal1 KO rat, the only other Bmal1 gene deletion model to show a diminished BP rhythm phenotype is the smooth muscle cell-specific KO model.^17^ These investigators reported an impaired BP rhythm with a nighttime decrease of systolic BP. All of these investigations were under ad libitum feeding conditions. Thus, further work is necessary to understand the interactions of the peripheral clocks with physiological responses to food intake and the regulation of BP rhythms. In addition to Bmal1, several other circadian genes have unique BP rhythms distinct from genetic controls. The Gumz laboratory has published a line of work demonstrating an important role for circadian gene Per1 in the regulation of BP and renal electrolyte excretion.^47^ This laboratory generated mice where Per1 was knocked out in two strains of mice (the salt-sensitive 129/SV and salt-resistant C57BL/6J) with distinct BP phenotypes. Deletion of Per1 in 129/SV mice led to a significantly lower BP compared with control mice; however, loss of Per1 in male C57BL/6 mice resulted in an increase in BP observed only during the active phase,^48^ and exhibited nondipping hypertension following high salt plus mineralocorticoid treatment. Interestingly, female Per1KO mice did not exhibit this nondipping hypertension phenotype following the same treatment.^10^ It would be interesting to determine what happens when RF was combined with dietary salt intervention.

Overall, our studies used a model of circadian misalignment by showing that timing of food intake entrains the diurnal BP rhythm in the mouse, independent of the light–dark cycle, or the core clock gene *Bmal1*. Although there is a great deal about this mechanism that is not known, it is tempting to speculate that the RF protocols in humans that report lower BP could be attributed to the lack of metabolic demands on the kidney and other organs during sleep. Furthermore, it will be important to discern how food consumption late at night or during the night may contribute to the phenomenon of nondipping BP and/or nocturnal hypertension. It is important to note, however, that in “real-life” conditions for humans such as chronic kidney disease or shift work may present as a combination of multiple factors, which may present difficulties to specifically determine the effect of RF on circadian BP rhythm.^49^ In addition, many time RF studies carried out in humans were often accompanied by calorie restriction.^50^^,^^51^ Sutton et al. demonstrated the positive effects of TRF (without calorie restriction or weight loss) on BP, yet the rhythmicity of BP remained unknown.^20^ Investigations in line with these concepts will be important for understanding hypertension risk and most likely target organ pathologies linked to loss of circadian rhythms.

## Supplementary Material

zqaa034_Supplementary_Data
