# Supplementary material for: Timing of Food Intake Drives the Circadian Rhythm of Blood Pressure
Source: Function (Oxf). 2020 Nov 24;2(1):zqaa034. doi: 10.1093/function/zqaa034 (PMC7772288; doi:10.1093/function/zqaa034)
Supplement: zqaa034_Supplementary_Data [file zqaa034_supplementary_data.pptx]

## Slide 1
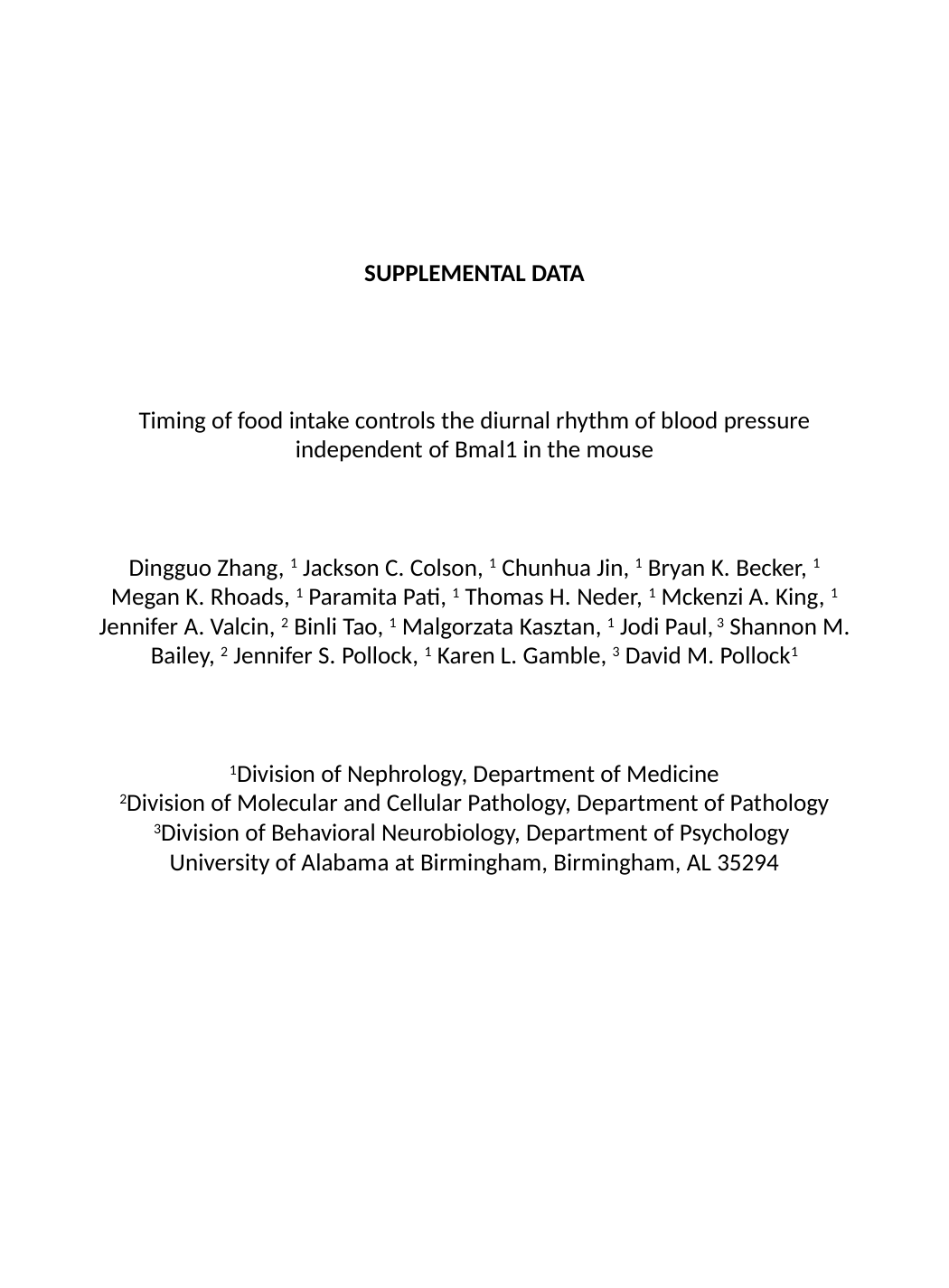

SUPPLEMENTAL DATA
Timing of food intake controls the diurnal rhythm of blood pressure independent of Bmal1 in the mouse
Dingguo Zhang, 1 Jackson C. Colson, 1 Chunhua Jin, 1 Bryan K. Becker, 1 Megan K. Rhoads, 1 Paramita Pati, 1 Thomas H. Neder, 1 Mckenzi A. King, 1 Jennifer A. Valcin, 2 Binli Tao, 1 Malgorzata Kasztan, 1 Jodi Paul, 3 Shannon M. Bailey, 2 Jennifer S. Pollock, 1 Karen L. Gamble, 3 David M. Pollock1
1Division of Nephrology, Department of Medicine
2Division of Molecular and Cellular Pathology, Department of Pathology
3Division of Behavioral Neurobiology, Department of Psychology
University of Alabama at Birmingham, Birmingham, AL 35294

## Slide 2
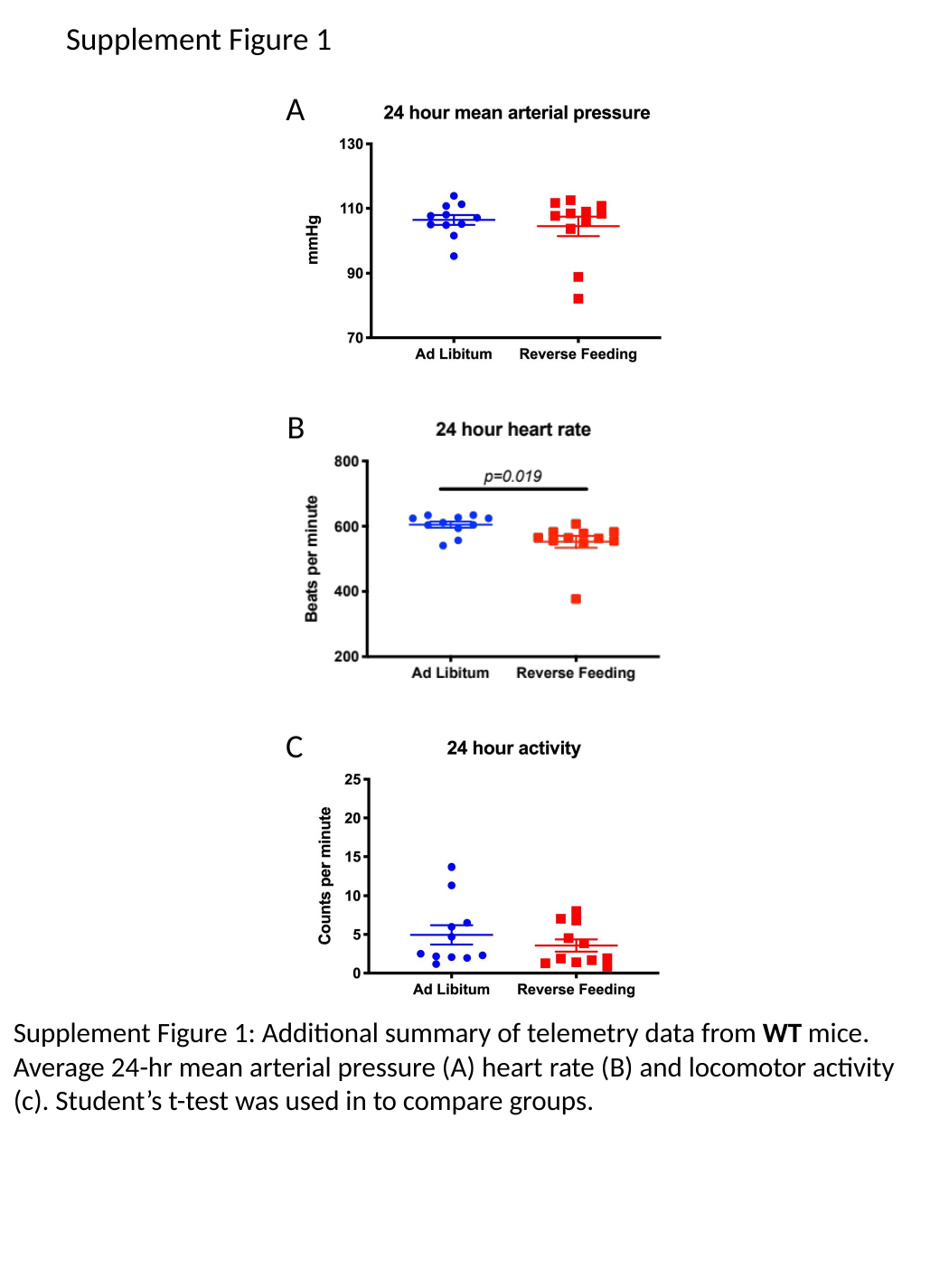

Supplement Figure 1
A
B
C
Supplement Figure 1: Additional summary of telemetry data from WT mice. Average 24-hr mean arterial pressure (A) heart rate (B) and locomotor activity (c). Student’s t-test was used in to compare groups.

## Slide 3
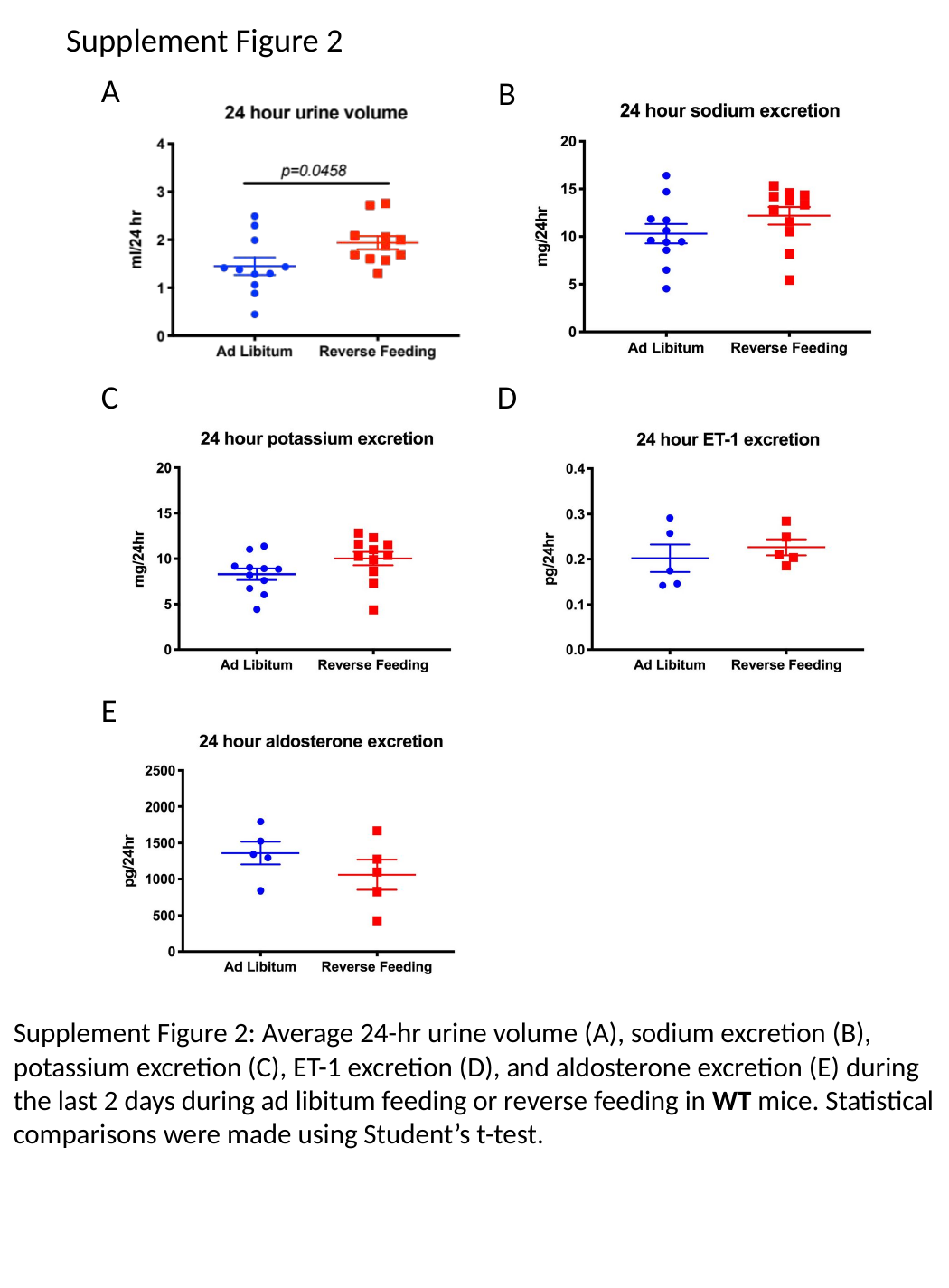

Supplement Figure 2
A
B
C
D
E
Supplement Figure 2: Average 24-hr urine volume (A), sodium excretion (B), potassium excretion (C), ET-1 excretion (D), and aldosterone excretion (E) during the last 2 days during ad libitum feeding or reverse feeding in WT mice. Statistical comparisons were made using Student’s t-test.

## Slide 4
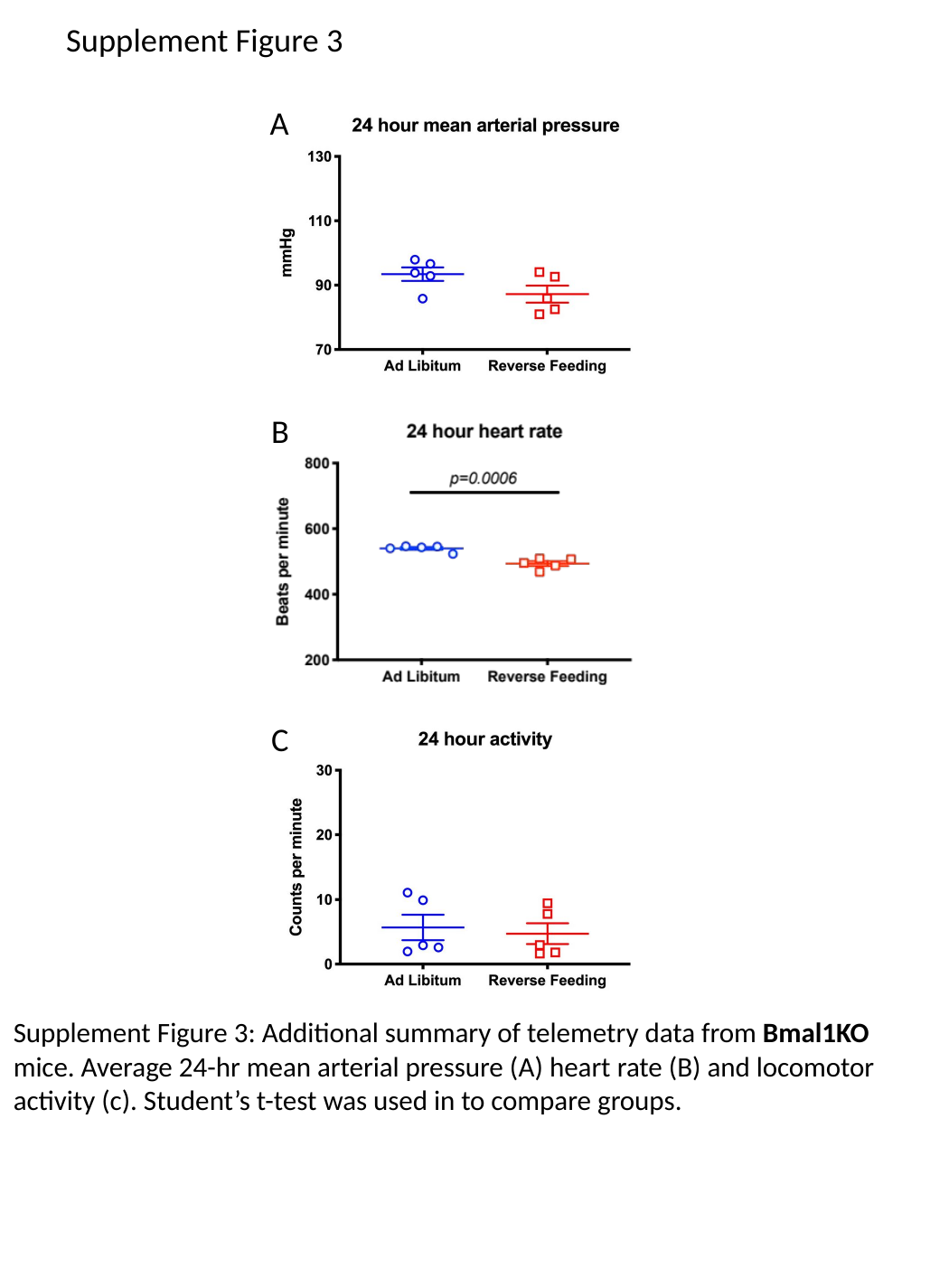

Supplement Figure 3
A
B
C
Supplement Figure 3: Additional summary of telemetry data from Bmal1KO mice. Average 24-hr mean arterial pressure (A) heart rate (B) and locomotor activity (c). Student’s t-test was used in to compare groups.

## Slide 5
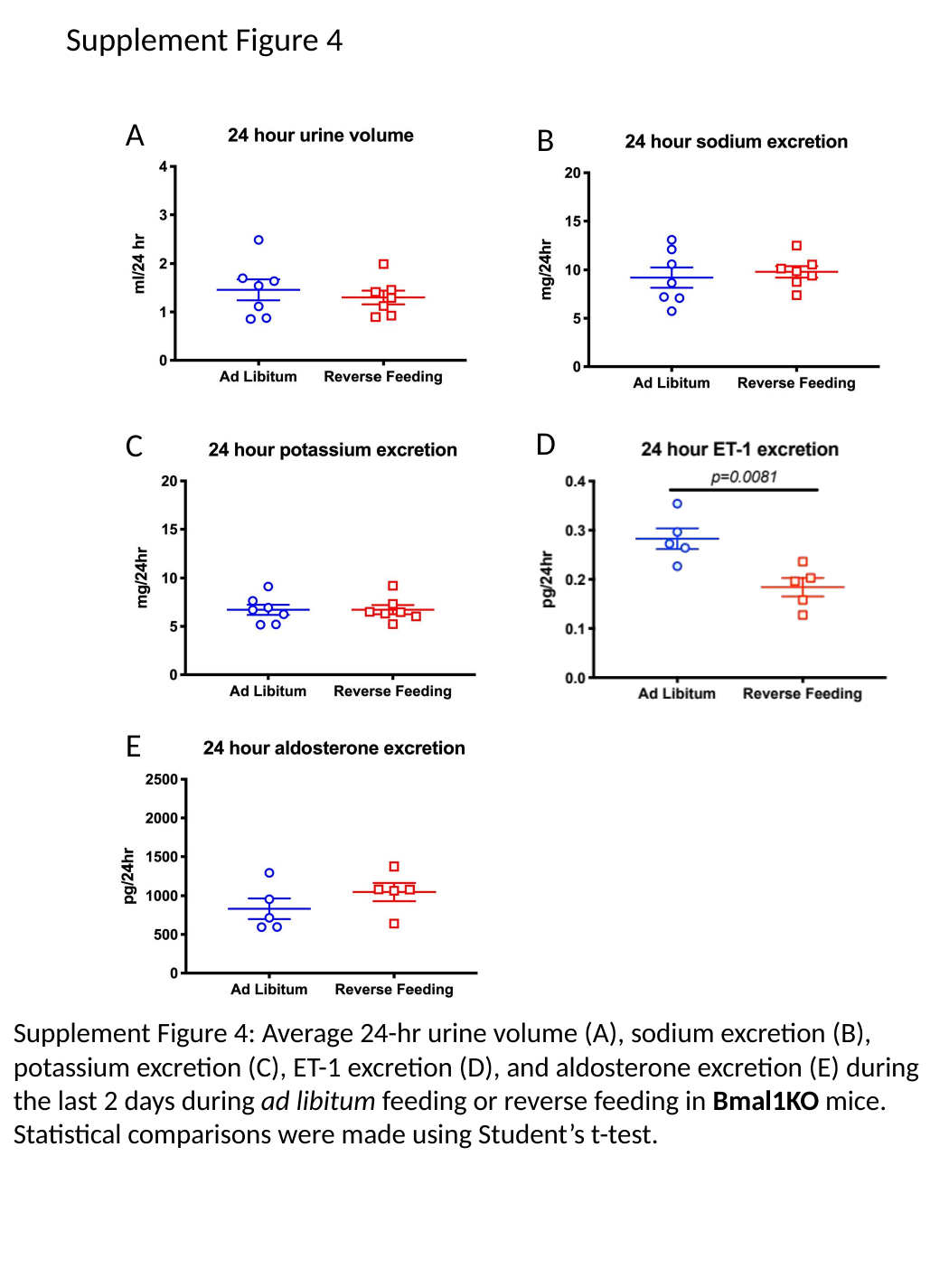

Supplement Figure 4
A
B
D
C
E
Supplement Figure 4: Average 24-hr urine volume (A), sodium excretion (B), potassium excretion (C), ET-1 excretion (D), and aldosterone excretion (E) during the last 2 days during ad libitum feeding or reverse feeding in Bmal1KO mice. Statistical comparisons were made using Student’s t-test.

## Slide 6
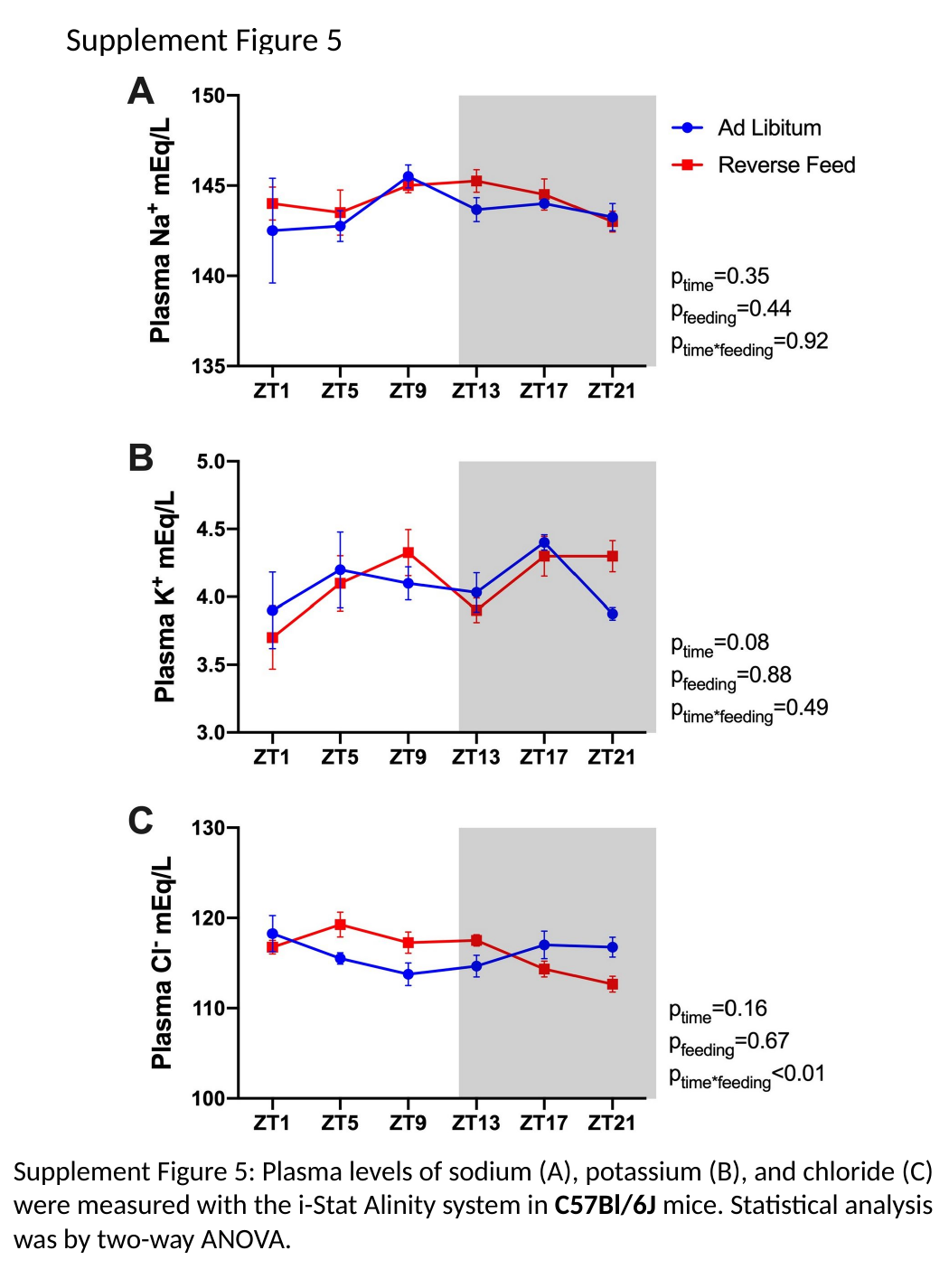

Supplement Figure 5
Supplement Figure 5: Plasma levels of sodium (A), potassium (B), and chloride (C) were measured with the i-Stat Alinity system in C57Bl/6J mice. Statistical analysis was by two-way ANOVA.

## Slide 7
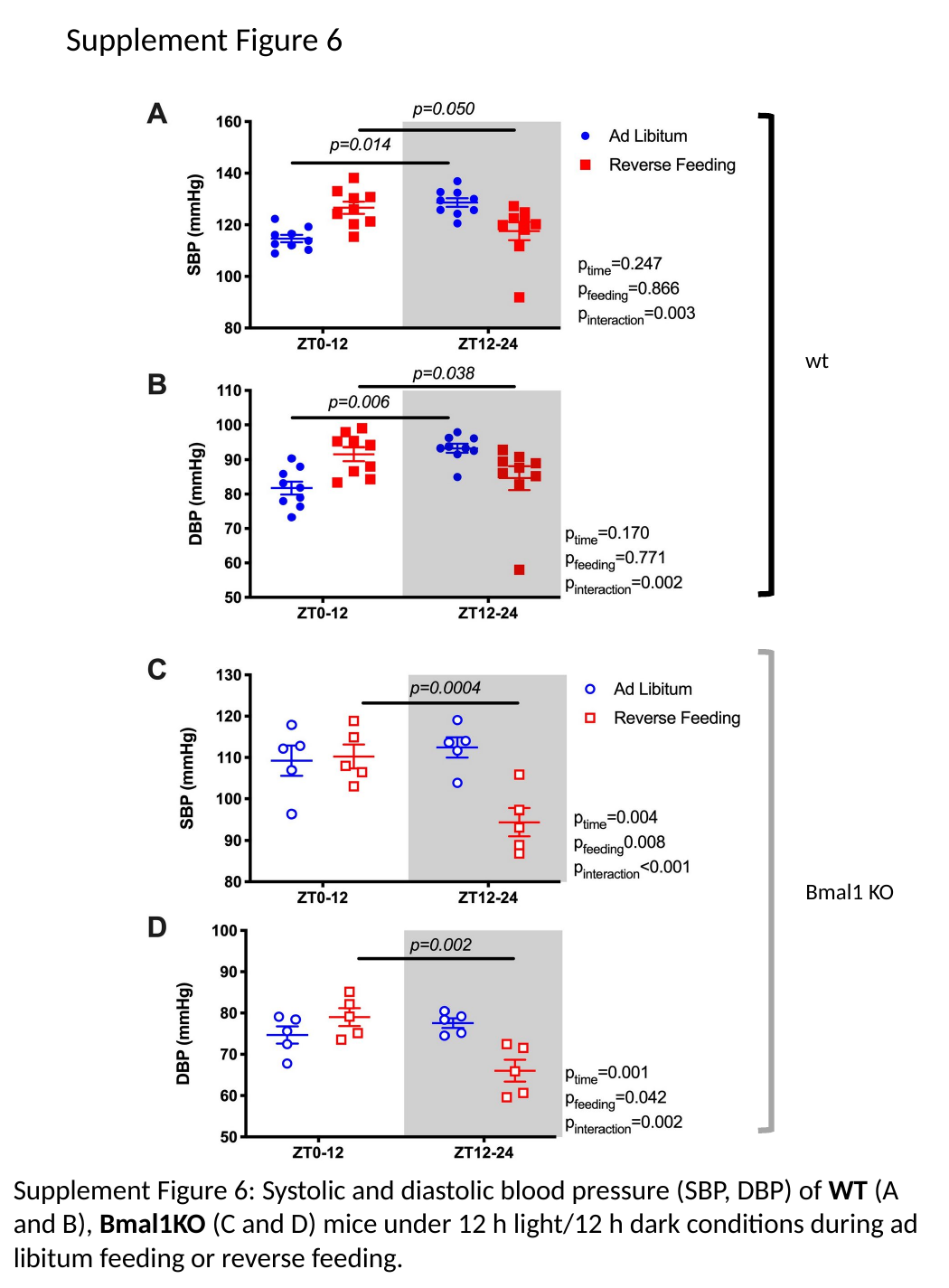

Supplement Figure 6
wt
Bmal1 KO
Supplement Figure 6: Systolic and diastolic blood pressure (SBP, DBP) of WT (A and B), Bmal1KO (C and D) mice under 12 h light/12 h dark conditions during ad libitum feeding or reverse feeding.

## Slide 8
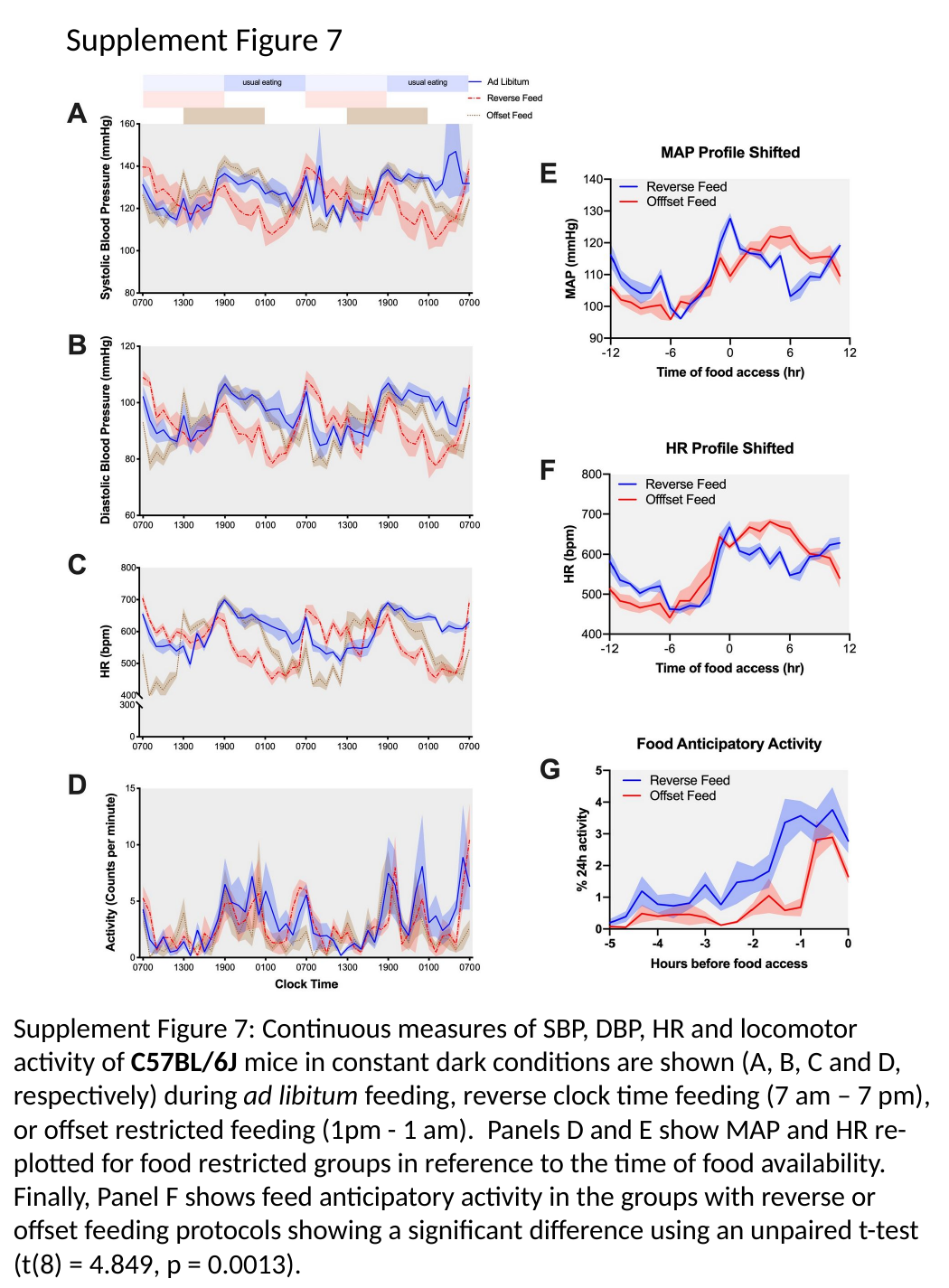

Supplement Figure 7
Supplement Figure 7: Continuous measures of SBP, DBP, HR and locomotor activity of C57BL/6J mice in constant dark conditions are shown (A, B, C and D, respectively) during ad libitum feeding, reverse clock time feeding (7 am – 7 pm), or offset restricted feeding (1pm - 1 am). Panels D and E show MAP and HR re-plotted for food restricted groups in reference to the time of food availability. Finally, Panel F shows feed anticipatory activity in the groups with reverse or offset feeding protocols showing a significant difference using an unpaired t-test (t(8) = 4.849, p = 0.0013).
